# Sexually transmitted infections knowledge in different populations attending a French university hospital: a prospective observational study

**DOI:** 10.1017/S0950268821000881

**Published:** 2021-04-21

**Authors:** Dulce Alfaiate, Susanna Giaché, Pierre Pradat, Laurent Cotte, Christian Chidiac, the Croix-Rousse STI Study Group, Florence Ader, Carole Adouard-Groslafeige, Agathe Becker, Evelyne Braun, Christine Fernandez, Tristan Ferry, Matthieu Godinot, Joanna Lippman-Trotignon, Aymeric Pansu, Thomas Perpoint, Sandrine Roux, Isabelle Schlienger, Claire Triffault-Fillit, Florent Valour, Fatima Yassir-Oria

**Affiliations:** 1 Service des Maladies Infectieuses et Tropicales, Hôpital de la Croix Rousse, Hospices Civils de Lyon, Lyon, France; 2 Service des Maladies Infectieuses et Tropicales, Centre Hospitalier Régional Orléans La Source, Orléans, France; 3 Centre de Recherche Clinique, Hôpital de la Croix Rousse, Hospices Civils de Lyon, Lyon, France; 4 Université Claude Bernard Lyon 1, Lyon, France

**Keywords:** HIV infection, PrEP, prevention, sexually transmitted infections, transmission

## Abstract

We conducted a prospective study about sexually transmitted infections (STIs) knowledge in different populations attending Lyon's University Hospitals in order to estimate awareness on STIs. Pre-exposure prophylaxis (PrEP)-users (PrEP group), persons living with HIV (PLWH group) and persons undergoing free STI screening (screening group) filled an anonymous questionnaire evaluating STI knowledge. A composite STI knowledge score was calculated and was correlated with patients’ characteristics. A total of 756 patients were enrolled in three groups: screening (*n* = 509), PrEP (*n* = 103) and PLWH (*n* = 144). STI transmission knowledge was better for HIV than for other STIs. The median STI knowledge score was significantly higher in PrEP-users than in the screening and PLWH groups. PrEP use and a previous STI diagnosis were independently associated with a higher score. PrEP-users have better STI knowledge than PLWH and persons undergoing free STI screening. Sexual health promotion interventions routinely reserved to PrEP-users in France seem to be effective in raising the awareness of this group for STIs. Continuous efforts are justified for PLWH and the younger layers of the population.

## Introduction

Sexually transmitted infections (STIs) are a major public health problem worldwide. They include a vast number of different pathogens transmitted through sexual contact causing both curable – as chlamydia, gonorrhoea, syphilis and hepatitis C – and incurable diseases – as hepatitis B, infection by the human immunodeficiency virus (HIV), herpes simplex virus and papillomavirus (HPV) infections. Hepatitis A and B, as well as HPV infections are preventable by vaccination.

The World Health Organization (WHO) estimates that more than 1 million curable STIs are acquired each day worldwide, and the burden of asymptomatic cases cannot but underestimate the problem [1]. Over the last 15 years, STI incidence has dramatically increased in many middle- and high-level income countries, including the USA and France [2, 3].

In France, STI diagnosis and treatment can be provided at any level of the health system and are reimbursed by the national health insurance. Additionally, a system of widely accessible STI clinics (designated ‘CeGIDD’) provides free and anonymous STI screening and treatment as well as vaccination and sexual health counselling [4]. Regular STI screening is also provided during the follow-up of persons living with HIV (PLWH) and persons receiving HIV pre-exposure prophylaxis (PrEP) [5].

As recognised by the WHO, counselling is an important primary prevention tool against STIs, especially in high-risk populations as men who have sex with men (MSM), sex workers and people who inject drugs [1]. Understanding general awareness of STIs is essential to develop and target effective sexual health interventions.

In order to identify the populations that would benefit most from sexual health campaigns, we conducted a prospective study about STI knowledge in different risk populations attending Lyon's University Hospitals.

## Methods

### Questionnaire development and validation

We developed a questionnaire including individual characteristics (demographic data, sexual orientation, number of sexual partners in the previous 6 months, systematic condom use, chemsex use and history of STIs) and questions aiming to evaluate patient knowledge on the clinical features, transmission, treatment and prevention of a group of STIs (HIV infection, hepatitis A, B and C, syphilis, gonorrhoea, chlamydia and HPV infections). The questionnaire face validity was ascertained by the members of the research group and clinical team and a pilot study was conducted to ascertain understandability.

The English version of the validated questionnaire is detailed in Supplementary Table S1.

### Patient recruitment

Patients were consecutively recruited at the Infectious Diseases Department of Croix-Rousse Hospital, Lyon, France between June and July 2018 and belonged to three groups: (1) persons attending the free STI clinic for screening (screening group); (2) PrEP-users (PrEP group); (3) PLWH group. Patients in the latter two groups were attending their regular follow-up visits.

The study was approved by the Institutional Review Board of the Lyon University Hospitals (approval number 18-07) and oral informed consent was obtained for all patients, after providing oral and written information on the study.

### Data collection

Patients were asked to anonymously complete the validated questionnaire and were excluded if they were unable to read and understand at least one of the languages used (French, English or Spanish).

Questionnaire completion was followed by an assessment of the answers with the referent doctor or nurse. Feedback on the correct answers and counselling were provided to the patients both verbally and in writing.

### Data analysis

For the calculation of unweighted composite STI knowledge score (ranging from −13 to +13), each correct answer was given one point and one point was subtracted if one or more misconceptions were present. As detailed in Supplementary Table S2, an answer was considered *correct* if all the true options were simultaneously indicated and a *misconception* was considered when any incorrect statement was selected.

Descriptive statistics were used to evaluate both demographic characteristics and individual answers to the questionnaire. Categorical data are presented as numbers and percentages and continuous data as medians and interquartile ranges (IQR25–IQR75). Frequencies of categorical variables were compared by χ^2^ test and continuous variables were compared by Kruskal–Wallis test.

Univariate analyses were performed using a linear regression model to identify factors potentially associated with STI knowledge score and variables with *P* < 0.05 as well as those known or suspected to be associated with the outcome were subsequently included in a multivariate model. Collinearity between variables was measured using the variance inflation factors (VIF) and a focused principal component analysis was performed to obtain a representation of the correlations between the outcome variable (STI knowledge score) and all other variables [6]. The relative importance of regressors in the linear model was measured using the lmg metrics (R package ‘relaimpo’) calculating R2 contribution averaged over orderings among regressors [7]. All the analyses were performed considering the overall patient population and later restricted to the MSM patients in each study group.

All analyses were performed using R (R Foundation for Statistical Computing, Vienna, Austria). Graphs were computed with GraphPad.

## Results

### Overall population

A total of 756 patients were enrolled (screening *n* = 509; PrEP *n* = 103 and PLWH *n* = 144). Significant differences between study groups were identified for all analysed characteristics as detailed in Table 1.

**Table 1.** Patients’ characteristics

	Screening (*n* = 509)	PrEP-users (*n* = 103)	PLWH (*n* = 144)	Total (*N* = 756)	*P* value^a^
Age, median [IQR]	24 [15–30]	36 [20–43.5]	47.5 [21–57]	28 [21–57]	<0.001
Male	308 (60.5)	103 (100)	115 (79.9)	523 (69.2)	<0.001
MSM	107 (21)	103 (100)	83 (57.6)	293 (38.8)	<0.001
Number of sexual partners in the previous 6 months					
1	162 (31.8)	1 (1.0)	91 (63.2)	254 (33.6)	<0.001
>1	347 (68.2)	102 (99)	53 (36.8)	502 (66.4)	<0.001
Systematic condom use	186 (36.5)	17 (16.5)	86 (59.7)	289 (38.2)	<0.001
With occasional partners^b^	106/292 (36.3)	17/100 (17%)	25/54 (46.3)	118/446 (33.2)	<0.001
Chemsex use	76 (14.9)	43 (41.7)	22 (15.3)	142 (18.8)	<0.001
Previous STI (other than HIV)					
No	387 (76.0)	17 (16.5)	41 (28.5)	445 (58.9)	<0.001
⩾1 STI	121 (23.8)	86 (83.5)	103 (71.5)	311 (41.1)	<0.001

Data are presented as numbers and percentages unless otherwise specified.

a Computed by *χ*^2^ for comparison of frequencies and Kruskal–Wallis for comparison of continuous variables.

b Denominator corresponds to the number of patients reporting occasional partners.

The median age ranged between 24 years in subjects undergoing screening and 47.5 years in PLWH. Male gender was dominant in all study groups (69% of all patients) and constituted 100% of the PrEP group. MSM represented 21% of the subjects undergoing screening and were majoritarian in the PLWH and PrEP groups (58% and 100%, respectively).

PLWH reported the highest rates of systematic condom use and lowest frequency of multiple sexual partners in the previous 6 months (60% and 37%, respectively). PrEP users had the lowest rate of systematic condom use (16%) and more frequently reported multiple sexual partners in the previous 6 months (99%) as well as higher rates of chemsex use and previous STIs (42% and 84%, respectively). Systematic condom use was reported in only 36% of the subjects undergoing STI screening. As the group not reporting systematic condom use may be overestimated by including individuals that do not systematically use a condom because they only have sex with their regular partner, we restricted the analysis to the individuals reporting sex with occasional partners (*n* = 446) and obtained similar proportions of systematic condom use (36.3% in the screening group, 17% in PrEP-users and 46.3% in PLWH).

The presence of a previous STI was reported by 41% of all the study subjects and ranged between 24% in subjects undergoing screening and 84% in PrEP-users.

The overall survey results are detailed in Supplementary Table S3.

Almost all patients (99%) answered questions regarding HIV modes of transmission, while answers for other STIs ranged from 62% (for gonorrhoea) to 80% (for syphilis). Overall, 86% of respondents were able to correctly indicate the modes of transmission for HIV infection, with statistically significant differences between the study groups (highest proportion among PrEP users). Knowledge of other STIs transmission was poor in all subgroups, with less than half of the subjects being able to correctly identify their modes of transmission (ranging from 16% for hepatitis A to 47% for syphilis). PrEP-users answered more frequently and had a better knowledge than other patients, regardless of the STI (from 42% for hepatitis A to 78% for syphilis and 80% for gonorrhoea).

Misconceptions regarding transmission of hepatitis A were reported by the majority of the patients (56% of the whole study patients, 65% of the PrEP users) and were rare for gonorrhoea and chlamydia (6% and 9% of the study population, respectively).

Less than 50% of patients correctly responded that most STIs are asymptomatic (74% among PrEP users). Almost 55% of the PLWH associated the absence of symptoms with an immune system deficiency. Overall, 80% of the study subjects recognised the risk of STI transmission from asymptomatic patients and 84% the risk of reinfection. The availability of effective treatments for hepatitis C, syphilis, gonorrhoea and chlamydia was recognised by 36% of respondents (64% of PrEP-users). Most patients were aware of the availability of vaccines for hepatitis A and HPV (62% and 52%), but 43% considered that hepatitis C could also be prevented through vaccination.

**Fig. 1.**
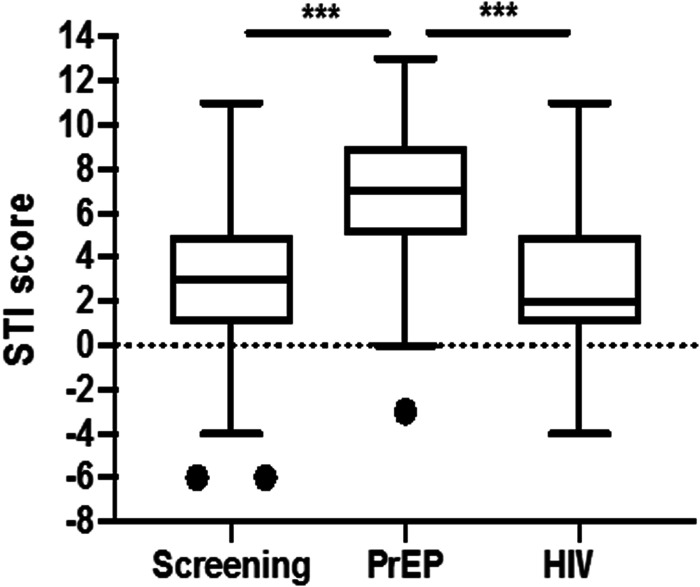
STI knowledge score comparison among study groups. Represented are the median value (central line), the 25th and 75th percentiles +/–1.5 IQR (box and whiskers’ limits, respectively). Values beyond these upper and lower bounds are considered outliers (dots). The *P* values are represented according to the following convention: *P* > 0.05 (non-significant, n.s.); *P* < 0.05 (*); *P* < 0.01 (**); *P* < 0.001 (***).

Overall, the median STI knowledge score was 3 [1–5] and was significantly higher in PrEP users (7 [5–9]) in comparison to other groups (3 [1–5] in the screening group and 2 [1–5] in PLWH, *P* < 0.001; Fig. 1).

The univariate analysis identified a significant association between an elevated STI knowledge score and male gender, sexual orientation (MSM), higher age, higher number of sexual partners, non-systematic condom use (overall and for individuals reporting sex with occasional partners), chemsex and a history of previous STI. In the subsequent multivariate analysis, only sexual orientation (MSM), PrEP use and the presence of a previous STI were significantly associated with a higher STI knowledge score (overall significance of the model gives *P* < 0.0001) (Table 2 and Supplementary Table S4). All VIF were below 3, indicating the absence of problematic correlation between variables. Focused principal component analysis is presented in Supplementary Figure S1.

**Table 2.** Univariate and multivariate analyses of factors potentially associated with STI knowledge score – overall study population

	Univariate analysis		Multivariate analysis^a^	
Variable	Estimate	*P* value	Estimate	*P* value
Gender (M)	0.7	0.008	−0.6	0.05
Sexual orientation (MSM)	2.1	<0.001	0.8	0.03
Risk group (screening *vs*. PrEP)	−4.1	<0.001	−2.6	<0.001
Risk group (PLWH *vs*. PrEP)	−4.4	<0.001	−3.4	<0.001
Age (per year)	0.02	0.04	0.008	0.5
Number of partners	1.1	<0.001	0.3	0.08
Systematic condom use^b^	−0.8	0.002	−0.09	0.7
Chemsex	1.2	<0.001	0.2	0.7
Previous STI	1.3	<0.001	0.6	0.006

a Overall significance of the model: *P* < 0.001.

b Univariate analysis restricted to individuals reporting occasional partners: estimate −0.95; *P* value 0.01.

### MSM

To account for the potential bias of sexual orientation, the analysis was further restricted to MSM (screening *n* = 107, PrEP *n* = 103, PLWH *n* = 83). The differences found in the overall study population were confirmed in this subset of patients, with a better identification of the modes of transmission of STIs and less frequent misconceptions among PrEP-users, in comparison to PLWH or persons undergoing STI screening (see Supplementary Table S5).

Although the median STI knowledge score was globally higher in this subset of patients than in the whole population (5 [2–7]), the differences previously found between subgroups were confirmed, with a significantly higher score among PrEP users (7 [5–10]) in comparison to other groups (4 [1–6] in the screening group and 3 [1–5] in PLWH).

Multivariate analysis in MSM further confirmed the results obtained for the whole study population with a significant association of a higher STI knowledge score with PrEP use and a previous history of STIs (Table 3).

**Table 3.** Univariate and multivariate analyses of factors potentially associated with STI knowledge score – MSM only

	Univariate analysis		Multivariate analysis^a^	
Variable	Estimate	*P* value	Estimate	*P* value
Risk group (screening *vs*. PrEP)	−3.3	<0.001	−2.0	<0.001
Risk group (PLWH *vs*. PrEP)	−4.0	<0.001	−3.2	<0.001
Age (per year)	−0.01	0.4	0.004	0.8
Number of partners	1.2	<0.001	0.4	0.07
Systematic condom use^b^	−1.5	<0.001	0.06	0.9
Chemsex	2.1	<0.001	0.8	0.1
Previous STI	1.4	<0.001	0.8	0.003

a Overall significance of the model: *P* < 0.001.

b Univariate analysis restricted to individuals reporting occasional partners: estimate −1.59; *P* value 0.003.

## Discussion

To the best of our knowledge, this is the first report comparing STI knowledge in different risk populations. In our setting, STI knowledge appears satisfactory for HIV but poorer for other STIs and the risk of transmission from asymptomatic patients is largely unrecognised. Regardless of the question, the best STI knowledge was identified among PrEP-users. In our population, this group was exclusively constituted by MSM and, in line with other cohorts, reported a significantly higher number of sexual partners, exposure to chemsex and history of STIs, as well as less frequent condom use [8].

STI awareness in the MSM population has been evaluated in recent studies, although with contradictory results, depending on the study population and the STI being evaluated (consistently better for HIV than for other STIs) [9, 10]. In our population, sexual orientation (MSM) was also significantly associated with a better STI awareness and could have been a potential bias in the comparison of PrEP-users to other groups. However, our survey first demonstrates that PrEP-users MSM have an STI knowledge score significantly higher than non-PrEP-users MSM, independently of a previous STI diagnosis, clearly demonstrating the impact of PrEP use on STI awareness. These data probably reflect the efficacy of educational programmes reserved to PrEP-users attending trimestral visits in our outpatient clinic. During all visits, patients meet a medical doctor and may also discuss with a nurse and a PrEP-counsellor (member of an association fighting STIs). As reported by the WHO, peer and lay counsellors can act as role models and offer non-judgemental and respectful support that can help to reduce stigma and facilitate access to services [11], and they may indeed play a key role in our PrEP follow-up programme.

Interestingly, PLWH, in spite of also attending regular follow-up visits, showed a significantly lower STI knowledge in comparison to PrEP users, in particular for STIs other than HIV. Their score was similar to the one observed in the general population undergoing sporadic STI screening, even though they reported a higher rate of systematic condom use and more frequent previous STI history. Although our analysis did not take into account the presence of efficient antiretroviral treatment or undetectable viral load, these findings, which are consistent with other reports [12], underline the need for specific STI assessment and counselling as part of routine HIV care.

Persons attending the STI clinic for sporadic screening were significantly younger than the two other study groups (median age 24 years). While the majority (75%) reported no previous STIs, only 36% declared to systematically use a condom with occasional partners, a low proportion that is in line with the findings of other European cohorts of the same age range [13,14]. The sporadic nature of the interactions with the STI clinic can partially justify a lower STI knowledge in this group than in the other study groups (that attend regular follow-up visits), but it is nonetheless clear that the overall STI awareness remains suboptimal in this population. Although adolescents and young adults are particularly at risk of contracting and transmitting STIs and suffering from long-term sequelae if not correctly oriented [15], barriers persist in their access to reliable information [13]. A continuous effort is hence warranted and both the educational system and complementary strategies as web-based and social marketing campaigns may have a role on the transmission of STI knowledge to this population [16–18].

Even though our study included an important number of subjects and allowed the identification of significant differences between different populations at risk of STI, limitations can be pointed out. Firstly, we acknowledge that the questionnaire has limitations that may preclude the interindividual comparability and its validation was limited by the absence of similar previously validated tools. Secondly, the voluntary nature of the questionnaire may have introduced a bias in the selection of patients and language and comprehension barriers may have prevented the inclusion of migrant individuals, a particularly at-risk population accounting for the majority of newly diagnosed HIV infections in France [19]. Moreover, the educational attainment of the included individuals was not taken into account in the analysis and may constitute a bias, given that PrEP-users in France have been shown to have a high instruction level [20]. Finally, this study was designed to evaluate the levels of STI knowledge that may not directly translate into a decrease in STI incidence. Indeed, the high incidence of STIs in well-informed populations such as PrEP users [21] underlines the need for further work to sustain the application of efficient preventive practices.

## Conclusions

STI transmission knowledge appears satisfactory for HIV but poorer for other STIs in all the analysed groups. Regardless of the STI being evaluated, the present study demonstrates that PrEP-users MSM have a significantly better STI knowledge compared to the other populations attending our outpatient clinic, including non-PrEP-users MSM. Sexual health promotion interventions routinely reserved to PrEP-users in France seem to be effective in raising the awareness of this group for STIs. Continuous efforts are justified for PLWH and the younger layers of the population and evaluation and promotion of STI awareness also among practitioners is warranted.

## Data Availability

The study material is available upon request to the corresponding author.

## Supplementary material

For supplementary material accompanying this paper visit http://dx.doi.org/10.1017/S0950268821000881. click here to view supplementary material
