# Supplementary material for: Sexually transmitted infections knowledge in different populations attending a French university hospital: a prospective observational study
Source: Epidemiol Infect. 2021 Apr 21;149:e105. doi: 10.1017/S0950268821000881 (PMC8193766; doi:10.1017/S0950268821000881)
Supplement: Supplementary file 1 [file S0950268821000881sup001.docx]

**Supplemental materials**

**Supplemental Table 1. STI knowledge questionnaire**

**Supplemental Table 2. STI knowledge score calculation**

**Supplemental Table 3. Questionnaire results per group – all patients**

**Supplemental Table 4. Relative contribution of predictive variables in the model**

**Supplemental Table 5. Questionnaire results per group – MSM only**

**Supplemental Figure 1. Focused Principal Component Analysis of the variables included in the multivariate model.**

**Supplemental Table 1. STI knowledge questionnaire**

Dear Visitor,

Thank you for taking the time to look our quiz. We would like to evaluate the knowledge of the persons that attend the hospital on sexual health and Sexually Transmitted Infections or STIs.

In order to do so we would be grateful if you would take the time to complete the following quiz, voluntarily and, of course, entirely anonymously. Your answers will help us to improve prevention and screening approaches for sexually transmitted infections.

The following questions are to better establish a demographic, all are optional, but it would help if you would be so kind as to answer as many as you feel comfortable with.

| Your age: | | years | | | You are: | | | | 🞏 | | | Trans | 🞏 | | Woman | | | 🞏 | Man | | |  |
| --- | --- | --- | --- | --- | --- | --- | --- | --- | --- | --- | --- | --- | --- | --- | --- | --- | --- | --- | --- | --- | --- | --- |
| Your visit today is for: | | | | | 🞏 | HIV/STI screening | | | 🞏 | | | HIV Follow-up | 🞏 | | PrEP | | | 🞏 | Other | | |  |
| Your sexual partners are: | | | | | 🞏 | Men | | | 🞏 | | | Women | 🞏 | | Both | | |  |  | | |  |
| Do you have a regular partner? | | | | | 🞏 | Yes | | | 🞏 | | | No |  | |  | | |  |  | | |  |
| Do you have occasional partners? | | | | | 🞏 | Yes | | | 🞏 | | | No |  | |  | | |  |  | | |  |
| How many different sexual partners have you had in the last 6 months? | | | | | | | | | | | | | | | | | | | | | |  |
| 🞏 | 1 only | | 🞏 | Between 1 - 5 | | | 🞏 | | | Between 5- 10 | | | | | | 🞏 | More than 10 | | | | |  |
| Do you use a condom: 🞏 Every time 🞏 Not every time  Do you ever use any recreational substances (drugs) during sex? | | | | | | | | | | | | | 🞏 | Yes | | | | | | 🞏 | No | |
| Have you ever had a sexually transmitted infections (STI) in the past? | | | | | | | | | | | | | | | | | | | | | |  |
| 🞏 | No, never | | 🞏 | Yes, once | | | | 🞏 | | | Yes, more than once | | | | | | | | | | |  |

**Table 1.** The following questions concern your knowledge about sexually transmitted infections. Are you aware of the transmission routes for the following infections? (Please check the boxes; there may be more than one possible answer)

|  | Vaginal sex | Anal sex | Oral sex (fellatio/  cunnilingus/  anilingus) | Sharing needles | Fisting | Sharing razors/ toothbrushes | Public toilets | Sharing sex toys | Saliva | Urine | Stools |
| --- | --- | --- | --- | --- | --- | --- | --- | --- | --- | --- | --- |
| HIV | € | € | € | € | € | € | € | € | € | € | € |
| Hepatitis A | € | € | € | € | € | € | € | € | € | € | € |
| Hepatitis B | € | € | € | € | € | € | € | € | € | € | € |
| Hepatitis C | € | € | € | € | € | € | € | € | € | € | € |
| Syphilis | € | € | € | € | € | € | € | € | € | € | € |
| Gonorrhea | € | € | € | € | € | € | € | € | € | € | € |
| Chlamydia | € | € | € | € | € | € | € | € | € | € | € |
| Genital warts/ HPV | € | € | € | € | € | € | € | € | € | € | € |

| **Question 1.** Do you think that all sexually transmitted infections have symptoms? | |
| --- | --- |
| 🞏 | 1. Yes, always. |
| 🞏 | 1. Yes, they often cause characteristic symptoms on the genitals or on the skin. |
| 🞏 | 1. Yes, if one knows the sexual habits of the person concerned. |
| 🞏 | 1. Yes, if there is a fever. |
| 🞏 | 1. No, almost never. |
|  | |
|  | |
| **Question 2.** If you have no symptoms of an STI (there may be more than one possible answer) | |
| 🞏 | 1. You probably have a very strong immune system that protects you against STI’s. |
| 🞏 | 1. You may be an asymptomatic carrier of the STI, without risk to you sexual partners. |
| 🞏 | 1. You may be an asymptomatic carrier of the STI, but with risk of infection to your sexual partners. |
| 🞏 | 1. In the absence of signs or symptoms your immunity ought to be tested. |
| 🞏 | 1. You could have a sexual health screening if you have had an episode at risk. |
|  | |
|  | |
| **Question 3.** For which of the following sexually transmitted is there an effective treatment? | |
| 🞏 | 1. Hepatitis C |
| 🞏 | 1. Syphilis |
| 🞏 | 1. Gonorrhea |
| 🞏 | 1. Chlamydia |
| 🞏 | 1. All of these infections |
|  | |
|  | |
| **Question 4.** After getting better from a STI can one get re-infected? | |
| 🞏 | 1. Yes, but only for Syphilis |
| 🞏 | 1. Yes, but only for Chlamydia |
| 🞏 | 1. Yes, but only for Gonorrhea |
| 🞏 | 1. Yes, for all STIs |
| 🞏 | 1. No, antibodies afford life-long protection |
|  | |
|  | |
| **Question 5.** Against which STIs do we have vaccinations? | |
| 🞏 | 1. Hepatitis A |
| 🞏 | 1. Hepatitis C |
| 🞏 | 1. Syphilis |
| 🞏 | 1. Gonorrhea |
| 🞏 | 1. HPV (Human Papillomavirus) |

**Supplemental Table 2. STI knowledge score calculation.** The STI knowledge score results from the sum of the number of points attributed to each question and can vary from -13 to +13.

|  | **+1 point if all of the following:** | **-1 point if one of the following:** |
| --- | --- | --- |
| **Table 1** |  |  |
| HIV | Vaginal, anal sex and needle sharing | Toilets, saliva, urine or stools |
| Hepatitis A | Stools | Vaginal, saliva or urine |
| Hepatitis B | Vaginal, anal, oral sex and needle sharing | Toilets, urine or stools |
| Hepatitis C | Anal sex and needle sharing | Oral, toilets, saliva, urine or stools |
| Syphilis | Vaginal, anal and oral sex | Toilets, urine or stools |
| Gonorrhea | Vaginal, anal and oral sex | Toilets |
| Chlamydia | Vaginal, anal and oral sex | Toilets |
| HPV | Vaginal, anal and oral sex | Toilets, saliva, urine or feces |
| **Questions** |  |  |
| Question 1(symptoms) | e | a, b, c or d |
| Question 2 (transmission) | c | a, b or d |
| Question 3 (treatment) | e | - |
| Question 4 (reinfection) | a | b, c, d or e |
| Question 5 (vaccine) | a or e | b, c or d |

**Supplemental Table 3. Questionnaire results per group – all patients**

|  | Screening (n=509) | PrEP-users (n=103) | PLWH (n=144) | Total (N=756) | P-value |  |
| --- | --- | --- | --- | --- | --- | --- |
| Modes of transmission (questionnaire table 1) | | | | | | |
| No answer |  |  |  |  |  |  |
| HIV, n (%) | 4 (1) | 1 (1) | 0 (0) | 5 (1) | 0.5 |  |
| Hepatitis A, n (%) | 181 (36) | 11 (11) | 57 (40) | 249 (33) | <0.001 |  |
| Hepatitis B, n (%) | 156 (31) | 12 (12) | 49 (34) | 217 (29) | <0.001 |  |
| Hepatitis C, n (%) | 161 (32) | 8 (8) | 46 (32) | 215 (28) | <0.001 |  |
| Syphilis, n (%) | 117 (23) | 4 (4) | 29 (20) | 150 (20) | <0.001 |  |
| Gonorrhea, n (%) | 227 (45) | 4 (4) | 58 (40) | 289 (38) | <0.001 |  |
| Chlamydia, n (%) | 136 (27) | 4 (4) | 58 (40) | 198 (26) | <0.001 |  |
| HPV, n (%) | 192 (38) | 12 (12) | 61 (42) | 265 (35) | <0.001 |  |
| Correct answers |  |  |  |  |  |  |
| HIV, n (%) | 437 (86) | 99 (96) | 115 (80) | 651 (86) | <0.001 |  |
| Hepatitis A, n (%) | 76 (15) | 43 (42) | 23 (16) | 142 (19) | <0.001 |  |
| Hepatitis B, n (%) | 139 (27) | 52 (50) | 37 (26) | 228 (30) | <0.001 |  |
| Hepatitis C, n (%) | 196 (39) | 64 (62) | 52 (36) | 312 (41) | 0.09 |  |
| Syphilis, n (%) | 209 (41) | 80 (78) | 66 (46) | 355 (47) | <0.001 |  |
| Gonorrhea, n (%) | 142 (28) | 82 (80) | 50 (35) | 274 (36) | <0.001 |  |
| Chlamydia, n (%) | 174 (34) | 79 (77) | 46 (32) | 299 (40) | <0.001 |  |
| HPV, n (%) | 145 (28) | 62 (60) | 35 (24) | 242 (32) | <0.001 |  |
| Misconceptions - HIV |  |  |  |  |  |  |
| HIV through toilets, n (%) | 30 (6) | 1 (1) | 3 (2) | 34 (4) | 0.03 |  |
| HIV through saliva, n (%) | 41 (8) | 2 (2) | 5 (3) | 48 (6) | 0.02 |  |
| HIV through urine, n (%) | 39 (8) | 5 (5) | 8 (6) | 52 (7) | 0.5 |  |
| HIV through stools, n (%) | 41 (8) | 12 (12) | 15 (10) | 68 (9) | 0.4 |  |
| ≥1 misconception, n (%) | 100 (20) | 15 (15) | 22 (15) | 137 (18) | 0.3 |  |
| Misconceptions- Hepatitis A |  |  |  |  |  |  |
| Hepatitis A through vaginal sex, n (%) | 257 (50) | 58 (56) | 55 (38) | 370 (49) | 0.001 |  |
| Hepatitis A through saliva, n (%) | 81 (16) | 26 (25) | 31 (22) | 138 (18) | 0.1 |  |
| Hepatitis A through urine, n (%) | 37 (7) | 14 (14) | 12 (8) | 63 (8) | 0.5 |  |
| ≥1 misconception, n (%) | 280 (57) | 67 (65) | 70 (49) | 417 (56) | 0.02 |  |
| Misconceptions - Hepatitis B |  |  |  |  |  |  |
| Hepatitis B through toilets, n (%) | 42 (8) | 3 (3) | 2 (1) | 47 (6) | 0.001 |  |
| Hepatitis B through urine, n (%) | 36 (7) | 12 (12) | 11 (1) | 59 (8) | 0.7 |  |
| Hepatitis B through stools, n (%) | 46 (9) | 18 (17) | 16 (11) | 80 (11) | 0.2 |  |
| ≥1 misconception, n (%) | 77 (15) | 20 (19) | 20 (14) | 117 (15) | 1 |  |
| Misconceptions - Hepatitis C |  |  |  |  |  |  |
| Hepatitis C through oral sex, n (%) | 166 (33) | 52 (50) | 40 (28) | 258 (34) | 0.15 |  |
| Hepatitis C through toilets, n (%) | 40 (8) | 3 (3) | 6 (4) | 49 (6) | 0.02 |  |
| Hepatitis C through saliva, n (%) | 73 (14) | 12 (12) | 19 (13) | 104 (14) | 0.2 |  |
| Hepatitis C through urine, n (%) | 30 (6) | 12 (12) | 8 (6) | 50 (7) | 0.4 |  |
| Hepatitis C through stools, n (%) | 43 (8) | 16 (16) | 15 (10) | 74 (10) | 0.4 |  |
| ≥1 misconception, n (%) | 196 (39) | 64 (62) | 52 (36) | 312 (41) | 0.09 |  |
| Misconceptions - Syphilis |  |  |  |  |  |  |
| Syphilis through toilets, n (%) | 38 (7) | 6 (6) | 8 (6) | 52 (7) | 0.3 |  |
| Syphilis through urine, n (%) | 47 (9) | 14 (14) | 9 (6) | 70 (9) | 0.4 |  |
| Syphilis through stools, n (%) | 38 (7) | 8 (8) | 8 (6) | 54 (7) | 0.6 |  |
| ≥1 misconception, n (%) | 73 (14) | 21 (20) | 19 (13) | 113 (15) | 0.7 |  |
| Misconceptions - Gonorrhea |  |  |  |  |  |  |
| Gonorrhea through toilets, n (%) | 32 (6) | 6 (6) | 5 (3) | 43 (6) | 0.14 |  |
| Misconceptions - Chlamydia |  |  |  |  |  |  |
| Chlamydia through toilets, n (%) | 55 (11) | 4 (4) | 11 (8) | 70 (9) | 0.02 |  |
| Misconception - HPV |  |  |  |  |  |  |
| HPV through toilets, n (%) | 106 (21) | 11 (11) | 15 (10) | 132 (17) | <0.001 |  |
| HPV through saliva, n (%) | 54 (11) | 6 (6) | 7 (5) | 67 (5) | 0.01 |  |
| HPV through urine, n (%) | 36 (7) | 10 (10) | 6 (4) | 52 (7) | 0.5 |  |
| HPV through stools, n (%) | 39 (8) | 8 (8) | 10 (7) | 57 (8) | 0.6 |  |
| ≥1 misconception, n (%) | 136 (27) | 20 (19) | 13 (9) | 169 (22) | <0.001 |  |
| Presence of symptoms of STIs (question 1) | | | | | | |
| Asymptomatic STIs, n (%) | 221 (43) | 76 (74) | 64 (44) | 361 (48) | <0.001 |  |
| Transmission of STIs if asymptomatic (question 2) | | | | | | |
| Risk of transmission /asymptomatic STI, n (%) | 421 (83) | 90 (87) | 96 (67) | 607 (80) | <0.001 |  |
| STI treatment (Hepatitis C, Syphilis, Gonorrhea, Chlamydia) (question 3) | | | | | | |
| All the above, n (%) | 149 (29) | 66 (64) | 58 (40) | 273 (36) | <0.001 |  |
| All the above except Hepatitis C, n (%) | 221 (43) | 96 (93) | 87 (60) | 404 (53) | <0.001 |  |
| Possibility of reinfection (question 4) | | | | | | |
| Reinfection, n (%) | 421 (83) | 100 (97) | 114 (79) | 635 (84) | <0.001 |  |
| Vaccine availability (question 5) | | | | | | |
| Hepatitis A, n (%) | 288 (57) | 95 (92) | 85 (59) | 468 (62) | <0.001 |  |
| Hepatitis C, n (%) | 234 (46) | 34 (33) | 60 (42) | 328 (43) | 0.05 |  |
| Syphilis, n (%) | 58 (11) | 7 (7) | 21 (15) | 86 (11) | 0.2 |  |
| Gonorrhea, n (%) | 31 (6) | 7 (7) | 10 (7) | 48 (6) | 0.9 |  |
| HPV, n (%) | 266 (52) | 67 (65) | 58 (40) | 391 (52) | <0.001 |  |
| Hepatitis A + HPV only, n (%) | 175 (34) | 63 (61) | 37 (26) | 275 (36) | <0.001 |  |
| STI knowledge score | | | | | | |
| Median (IQR) | 3 (1-5) | 7 (5-9) | 2 (1-5) | 3 (1-5) | <0.001 |  |

**Supplemental Table 4. Relative contribution of predictive variables in the model**

1. **Relative importance of predicting variables reported as contribution percentages**

| Risk group | 46.9% |
| --- | --- |
| Gender | 1.9% |
| Sexual orientation | 14.1% |
| Age | 0.1% |
| Number of partners | 17.9% |
| Chemsex | 2.6% |
| Previous STI | 15.9% |

1. **Average coefficients for different model sizes**

|  | 1X | 2Xs | 3Xs | 4Xs | 5Xs | 6Xs | 7Xs |
| --- | --- | --- | --- | --- | --- | --- | --- |
| Gender | 0.72 | 0.16 | -0.16 | -0.34 | -0.44 | -0.51 | -0.56 |
| Sexual orientation | 2.05 | 1.67 | 1.36 | 1.12 | 0.95 | 0.83 | 0.75 |
| Screening vs PreP | -4.10 | -3.66 | -3.33 | -3.07 | -2.86 | -2.70 | -2.56 |
| PLWH vs PreP | -4.37 | -4.10 | -3.89 | -3.73 | -3.61 | -3.53 | -3.46 |
| Age | 0.02 | 0.01 | 0.004 | 0.003 | 0.004 | 0.006 | 0.008 |
| Number of partners | 1.10 | 0.89 | 0.72 | 0.58 | 0.46 | 0.36 | 0.26 |
| Chemsex | 1.19 | 0.82 | 0.58 | 0.42 | 0.32 | 0.26 | 0.21 |
| Previous STI | 1.33 | 1.09 | 0.91 | 0.77 | 0.68 | 0.60 | 0.55 |

**Supplemental Table 5. Questionnaire results per group – MSM only**

|  | Screening (n=107) | PrEP-users (n=103) | PLWH (n=83) | Total (N=293) | P value | |
| --- | --- | --- | --- | --- | --- | --- |
| Modes of transmission (questionnaire table 1) | | | | | |  |
| No answer |  |  |  |  |  | |
| HIV, n (%) | 1 (1) | 1 (1) | 0 (0) | 2 (1) | 0.67 | |
| Hepatitis A, n (%) | 39 (27) | 11 (11) | 29 (35) | 79 (24) | <0.001 | |
| Hepatitis B, n (%) | 31 (22) | 12 (12) | 21 (25) | 64 (19) | 0.05 | |
| Hepatitis C, n (%) | 31 (22) | 8 (8) | 21 (25) | 60 (18) | 0.005 | |
| Syphilis, n (%) | 20 (14) | 4 (4) | 10 (12) | 34 (10= | 0.02 | |
| Gonorrhea, n (%) | 38 (27) | 4 (4) | 27 (32) | 69 (21) | <0.001 | |
| Chlamydia, n (%) | 21 (15) | 4 (4) | 27 (32) | 52 (16) | <0.001 | |
| HPV, n (%) | 37 (26) | 12 (12) | 27 (32) | 76 (23) | 0.003 | |
| Correct answers |  |  |  |  |  | |
| HIV, n (%) | 126 (89) | 99 (98) | 69 (82) | 294 (90) | 0.004 | |
| Hepatitis A, n (%) | 34 (24) | 43 (52) | 17 (47) | 94 (49) | 0.003 | |
| Hepatitis B, n (%) | 52 (37) | 52 (65) | 26 (55) | 130 (61) | 0.02 | |
| Hepatitis C, n (%) | 72 (51) | 64 (73) | 36 (76) | 172 (78) | 0.04 | |
| Syphilis, n (%) | 83 (58) | 80 (84) | 49 (75) | 212 (80) | 0.02 | |
| Gonorrhea, n (%) | 58 (41) | 82 (86) | 37 (96) | 177 (86) | <0.001 | |
| Chlamydia, n (%) | 69 (49) | 79 (83) | 33 (85) | 181 (78) | <0.001 | |
| HPV, n (%) | 56 (39) | 62 (77) | 22 (57) | 140 (72) | <0.001 | |
| Misconceptions - HIV |  |  |  |  |  | |
| HIV through toilets, n (%) | 6 (4) | 1 (1) | 0 (0) | 7 (2) | 0.02 | |
| HIV through saliva, n (%) | 11 (8) | 2 (2) | 2 (2) | 15 (5) | 0.04 | |
| HIV through urine, n (%) | 11 (8) | 5 (5) | 4 (5) | 20 (6) | 0.83 | |
| HIV through stools, n (%) | 12 (8) | 12 (12) | 9 (11) | 33 (10) | 0.95 | |
| ≥1 misconception, n (%) | 27 (19) | 15 (15) | 10 (12) | 52 (16) | 0.25 | |
| Misconceptions - Hepatitis A |  |  |  |  |  | |
| Hepatitis A through vaginal sex, n (%) | 85 (60) | 58 (56) | 35 (42) | 178 (54) | 0.03 | |
| Hepatitis A through saliva, n (%) | 26 (18) | 26 (25) | 20 (24) | 72 (22) | 0.81 | |
| Hepatitis A through urine, n (%) | 15 (11) | 14 (14) | 9 (11) | 38 (12) | 0.77 | |
| ≥1 misconception, n (%) | 91 (65) | 67 (65) | 44 (52) | 202 (62) | 0.15 | |
| Misconceptions - Hepatitis B |  |  |  |  |  | |
| Hepatitis B through toilets, n (%) | 15 (11) | 3 (3) | 1 (1) | 19 (6) | 0.004 | |
| Hepatitis B through urine, n (%) | 13 (9) | 12 (12) | 8 (10) | 33 (10) | 0.73 | |
| Hepatitis B through stools, n (%) | 17 (12) | 18 (17) | 10 (12) | 45 (14) | 0.52 | |
| ≥1 misconception, n (%) | 28 (20) | 20 (19) | 14 (17) | 62 (19) | 0.81 | |
| Misconceptions - Hepatitis C |  |  |  |  |  | |
| Hepatitis C through oral sex, n (%) | 61 (43) | 52 (50) | 29 (35) | 142 (43) | 0.10 | |
| Hepatitis C through toilets, n (%) | 14 (10) | 3 (3) | 4 (5) | 21 (6) | 0.07 | |
| Hepatitis C through saliva, n (%) | 26 (18) | 12 (12) | 12 (14) | 50 (15) | 0.09 | |
| Hepatitis C through urine, n (%) | 15 (11) | 12 (12) | 7 (8) | 34 (10) | 0.77 | |
| Hepatitis C through stools, n (%) | 20 (14) | 16 (16) | 10 (12) | 46 (14) | 0.72 | |
| ≥1 misconception, n (%) | 77 (54) | 61 (59) | 33 (39) | 171 (52) | 0.01 | |
| Misconceptions - Syphilis |  |  |  |  |  | |
| Syphilis through toilets, n (%) | 12 (8) | 6 (6) | 4 (5) | 22 (7) | 0.57 | |
| Syphilis through urine, n (%) | 20 (14) | 14 (14) | 5 (6) | 39 (12) | 0.13 | |
| Syphilis through stools, n (%) | 14 (10) | 8 (8) | 4 (5) | 26 (8) | 0.28 | |
| ≥1 misconception, n (%) | 28 (20) | 21 (20) | 8 (10) | 57 (17) | 0.07 | |
| Misconceptions - Gonorrhea |  |  |  |  |  | |
| Gonorrhea through toilets, n (%) | 11 (8) | 6 (6) | 3 (4) | 20 (6) | 0.39 | |
| Misconceptions - Chlamydia |  |  |  |  |  | |
| Chlamydia through toilets, n (%) | 19 (13) | 4 (4) | 6 (7) | 29 (9) | 0.03 | |
| Misconceptions - HPV |  |  |  |  |  | |
| HPV through toilets, n (%) | 34 (24) | 11 (11) | 11 (13) | 56 (17) | 0.02 | |
| HPV through saliva, n (%) | 19 (13) | 6 (6) | 5 (6) | 30 (9) | 0.06 | |
| HPV through urine, n (%) | 15 (11) | 10 (10) | 4 (5) | 29 (9) | 0.36 | |
| HPV through stools, n (%) | 16 (11) | 8 (8) | 6 (7) | 30 (9) | 0.56 | |
| ≥1 misconception, n (%) | 46 (32) | 20 (19) | 17 (20) | 83 (25) | 0.03 | |
| Presence of symptoms of STIs (question 1) | | | | | |  |
| Asymptomatic STIs, n (%) | 64 (45) | 76 (74) | 34 (40) | 174 (53) | <0.001 | |
| Transmission of STIs if asymptomatic (question 2) | | | | | |  |
| Risk of transmission /asymptomatic STI, n (%) | 119 (84) | 90 (87) | 54 (64) | 263 (80) | <0.001 | |
| STI treatment (Hepatitis C, Syphilis, Gonorrhea, Chlamydia) (question 3) | | | | | |  |
| All the above, n (%) | 39 (27) | 66 (64) | 34 (40) | 139 (42) | <0.001 | |
| All above except Hepatitis C, n (%) | 81 (57) | 96 (93) | 54 (64) | 231 (70) | <0.001 | |
| Possibility of reinfection (question 4) | | | | | |  |
| Reinfection, n (%) | 121 (85) | 100 (97) | 66 (79) | 287 (87) | <0.001 | |
| Vaccine availability (question 5) | | | | | |  |
| Hepatitis A, n (%) | 91 (64) | 95 (92) | 48 (57) | 234 (71) | <0.001 | |
| Hepatitis C, n (%) | 60 (42) | 34 (33) | 31 (37) | 125 (38) | 0.24 | |
| Syphilis, n (%) | 13 (9) | 7 (7) | 15 (18) | 35 (11) | 0.02 | |
| Gonorrhea, n (%) | 10 (7) | 7 (7) | 7 (8) | 24 (7) | 0.86 | |
| HPV, n (%) | 73 (51) | 67 (65) | 33 (39) | 173 (53) | 0.002 | |
| Hepatitis A + HPV only, n (%) | 50 (35) | 63 (61) | 21 (25) | 134 (41) | <0.001 | |
| STI knowledge score | | | | | |  |
| Median (IQR) | 4 (1-6) | 7 (5-10) | 3 (1-5) | 5 (2-7) | <0.001 | |

**Supplemental Figure 1. Focused Principal Component Analysis of the variables included in the multivariate model. Green dots indicate a positive correlation and yellow dots a negative correlation.**
